# Hemiarthroplasty of the hip using the direct anterior approach

**DOI:** 10.1007/s00064-021-00727-6

**Published:** 2021-08-03

**Authors:** Michael Nogler, Filippo Randelli, George A. Macheras, Martin Thaler

**Affiliations:** 1 Department of Orthopedic Surgery—Experimental Orthopedics, Medical University Innsbruck, Innrain 36, 6020 Innsbruck, Austria; 2 CAD—ASST Gaetano Pini-CTO, Centro Specialistico Ortopedico Traumatologico, P.za A. Ferrari, 1, 20122 Milan, Italy; 3 Department of Orthopedics, “KAT” General Hospital of Attica, Athens, Greece; 4 Department of Orthopedic Surgery, Medical University Innsbruck, Anichstr. 35, 6020 Innsbruck, Austria

**Keywords:** Reconstructive surgical procedures, Arthroplasty replacement, Femoral neck fractures, Acetabulum, Minimally invasive surgical procedures, Rekonstruktive chirurgische Verfahren, Endoprothesenersatz, Schenkelhalsfrakturen, Azetabulum, Minimalinvasive chirurgische Verfahren

## Abstract

**Objective:**

Minimally invasive approach in total hip arthroplasty for the treatment of femoral neck fractures with a hemiarthroplasty.

**Indications:**

Femoral neck fractures of patients without hip osteoarthritis where the acetabulum is still intact.

**Contraindications:**

Lesions and infections of the skin in the approach area; hip osteoarthritis; surgeon’s lack of experience with the technique.

**Surgical technique:**

The direct anterior approach (DAA) uses the Smith–Peterson interval between the tensor fasciae latae (TFL) and the rectus and sartorius muscle. After coagulation of the ascending branches of the femoral circumflex vessels, the capsule is opened. The remaining parts of the femoral neck are removed and osteotomized if necessary. The femoral head is removed with a cork screw. Then the shaft is supported by 2 sharp retractors at the greater trochanter from cranial, and the leg is externally rotated, hyperextended, and adducted. A TFL release can be performed which we also recommend. The femoral canal is opened step by step and extended with rasps which are introduced with the double curved broach handle. Cement and the final implant are introduced and after the trial reduction also the final head. The hip is reduced, the capsule adapted and the wound closed.

**Postoperative management:**

For this approach, there are no approach specific recommendations. Postoperative treatment depends on whether the approach was extended with muscle releases and on the type of reconstruction performed. If the approach was limited to the minimally invasive direct anterior portal, quicker rehabilitation can be expected due to the reduced muscle damage. We prefer mobilization with full weight bearing as tolerated on the next day.

## Abstract (de)

**Operationsziel:**

Der minimalinvasive Zugang ist muskelschonend, erlaubt eine rasche Operation und frühe Mobilisation.

**Indikationen:**

Indikationen für eine Versorgung mit einer Hemiarthroplastik sind Schenkelhalsfrakturen in Fällen ohne Koxarthrose, in denen das Azetabulum noch intakt ist.

**Kontraindikationen:**

Läsionen und Infektionen der Haut im Zugangsgebiet, Koxarthrose sowie die fehlende Erfahrung des Operateurs sind Kontraindikationen.

**Operationstechnik:**

Der direkte anteriore Zugang erfolgt über das Smith-Peterson-Intervall zwischen M. tensor fasciae latae (TFL) und M. rectus bzw. M. sartorius. Unter Koagulation der aufsteigenden Äste der Vasa circumflexa femoris wird die Kapsel dargestellt und eröffnet. Die freien Schenkelhalsreste werden entfernt, und eine glatte Osteotomie wird auf der gewünschten Höhe hergestellt. Der Hüftkopf wird mit einem Korkenzieher entfernt. Der Schaft wird mit einem 2‑spitzigen Heben am Trochanter major von kranial her unterstützt und das Bein in Außenrotation, Hyperextension und Adduktion gebracht. Zur Verbesserung der Exposition des Femureingangs kann ein TFL-Release erfolgen, was die Autoren empfehlen. Danach wird der Markkanal schrittweise eröffnet und mit Raspeln, die an einem doppelt kurvierten Handgriff befestigt sind, erweitert. Zement und das finale Implantat werden eingebracht und nach Probereposition auch der definitive Kopf. Danach wird die Hüfte reponiert, die Kapsel adaptiert und die Wunde verschlossen.

**Weiterbehandlung:**

Für diesen Zugang gibt es keine zugangsspezifischen Empfehlungen. Die postoperative Behandlung hängt von vorgenommenen Erweiterungen des Zugangs mit Muskelablösungen und der Art der erfolgten Rekonstruktion ab. Wenn der Zugang auf das minimalinvasive direkte anteriore Portal beschränkt war, ist durch den reduzierten Muskelschaden eine deutlich raschere Rehabilitation zu erwarten. Die Autoren empfehlen Mobilisation mit voller Belastung, falls erträglich, am nächsten Tag.

## Introductory remarks

The direct anterior approach to the hip was described as early as the 1880s by Carl Hueter [3] and was widely popularized for pelvic and pediatric surgery by Smith Petersen [19]. Pioneers of the early days like Keggi [5] or Judet et al. [4] used it for total hip arthroplasty. Yet only in the past 20 years has this approach been popularized as an approach with minimal muscle damage. The main advantage of the direct anterior approach (DAA) is preservation of the muscular structures, especially the gluteal muscles which remain intact during primary total hip arthroplasty (THA) through this approach. The treatment of femoral neck fractures in older and often multimorbid patients requires a time-saving and gentle surgical technique. The DAA fulfills all these requirements as no splitting or transection of the muscles is required and it can be quickly performed. The rehabilitation can start early and due to the minimal muscle trauma can be faster. It does have the potential to reduce 1‑year mortality according to a recent study [2]. The blood loss is very low [22]—as well as the dislocation rate [21]—and the patient can be easily remobilized due to little pain. Like total hip arthroplasty, hemiarthroplasty has become a successful standard procedure [8, 23].

## Surgical principle and objective

The direct anterior approach (DAA) to the hip in cases of hemiarthroplasty is muscle sparing. In many instances, with elaborate instrumentation and shorter implants it is possible to avoid any muscular releases; however, if the situation is more challenging, an additional release is still possible in our experience. Furthermore, to avoid damage of the greater trochanter in patients with osteoporotic bone, it is recommended to perform a release of the tensor fasciae latae (TFL) muscle. We regard the TFL as a muscle that is of minimal importance to hip motion. The release and repair technique we propose allows for a full repair of the muscle inside the iliotibial band and does not negatively influence early mobilization or long-term rehabilitation. The DAA technique allows rapid surgery and can be performed on a standard operating table with very little effort.

## Advantages

- Standard approach to the hip
- Preservation of the muscular structures especially gluteal abductional function
- Short skin incision
- Low blood loss [22]
- Faster rehabilitation compared to the anterolateral approach [10]
- Lower risk of dislocation compared with the posterior approach [21]
- A distal extension of the approach is possible in cases of femoral fractures [20]

## Disadvantages

- Smaller approach
- Technically more demanding
- Training and experience necessary
- Potential lesion of the lateral femoral cutaneous nerve
- Risk of femoral fractures [12]
- Specialized instruments are mandatory

## Indications

- Femoral neck fractures with no hip osteoarthritis

## Contraindications

- Skin infection in the approach area (intertriginous area)
- Osteomyelitis (general)
- Bacterial hip osteoarthritis (general)

## Patient information

- Injury to the lateral femoral cutaneous nerve (LFCN) with numbness at the distal lateral femur
- Meralgia paresthetica of the LFCN
- Skin scar at the front thigh

## Preoperative work up

- Treatment of infections in the intertriginous area

## Instruments and implants

**Fig. 1:**
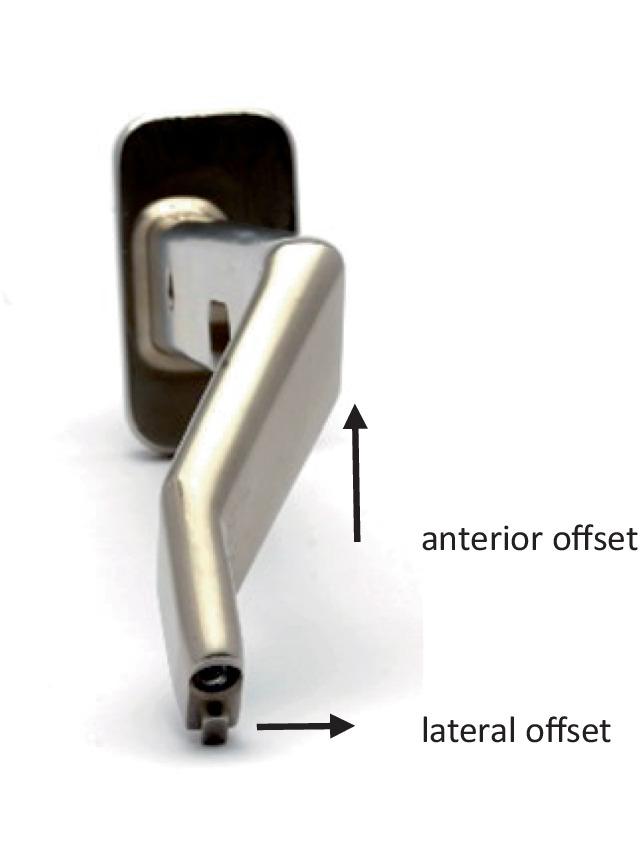
Broach handle with double-offset (anterior and lateral)

- Specially curved and angulated instruments
- Instruments with offset
- A special double-pronged elevator for the femur (Fig. 1)

## Anesthesia and positioning

- General and neuraxial anesthesia are both appropriate for this approach
- Positioning of the patient in supine position on the operating table
- Draping of both legs enables the operative leg to be crossed under the opposite leg during the exposure of the femur
- The table needs to be folded down at the level of the hip joint to flex the hip (exposure of the acetabulum) and extended (exposure of the femoral canal)
- If available and desired, use of a leg holder

## Surgical technique

(Figs. 2, 3, 4, 5, 6, 7, 8, 9, 10, 11, 12, 13, 14, 15, 16, 17, 18)

**Fig. 2:**
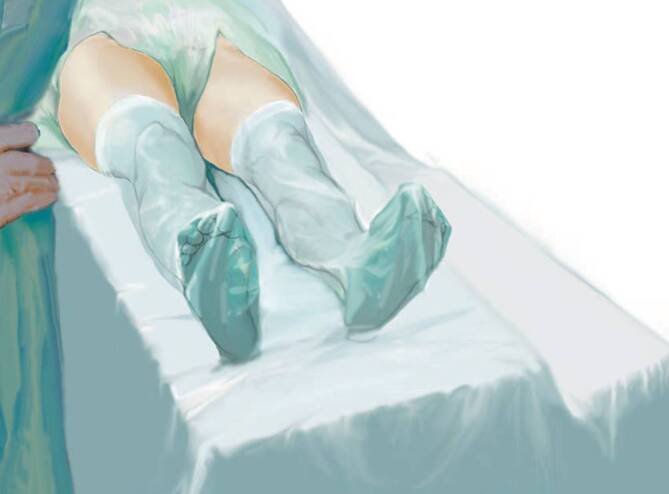
In hemiarthroplasty of the hip using the direct anterior approach (DAA), the patient is positioned in supine position on the operating table. This position keeps the hip in a stable position, and leg length can be easily measured. For the exposure of the acetabulum with the femoral component in situ, a table attachment for the lower leg at the operating table is necessary. A table attachment at the operating table (such as an arm board) facilitates hyperabduction of the opposite leg during exposure of the femur. Both legs are flexibly draped. This allows the operative leg to be crossed under the opposite leg during exposure of the femur. In addition, there are leg holders available on the market which can be sterile draped, which allows the surgeon to fix the leg holder at any position. With these leg holders, a stable position of the leg can be achieved. The leg holder can also replace one assistant. Also, the use of a retractor-holding system can replace one assistant. The reduction of the number of assistants to one is especially important for night shifts and is more relevant for the treatment of femoral neck fractures than for planned treatments with total endoprosthesis [1]. (From [14])

**Fig. 3:**
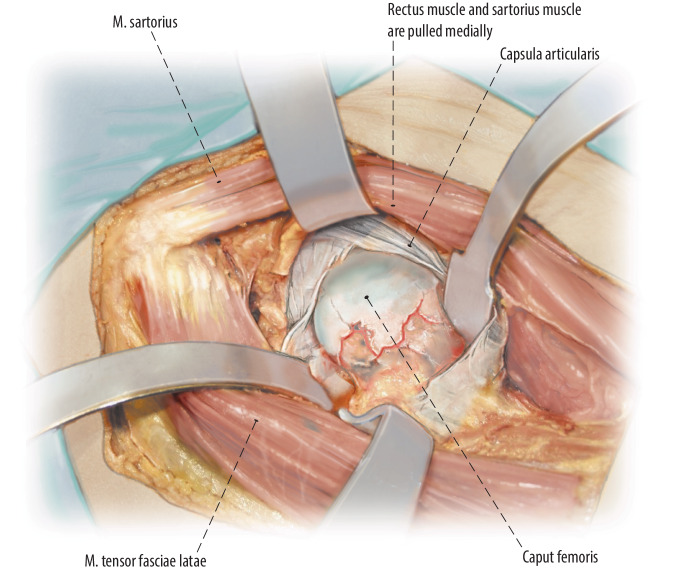
The approach is located between the tensor fasciae latae muscle and the sartorius muscle/rectus femoris muscle. If distal extension is needed, the skin incision can be extended in a “Lazy S” shape as described elsewhere [6, 14, 15]

**Fig. 4:**
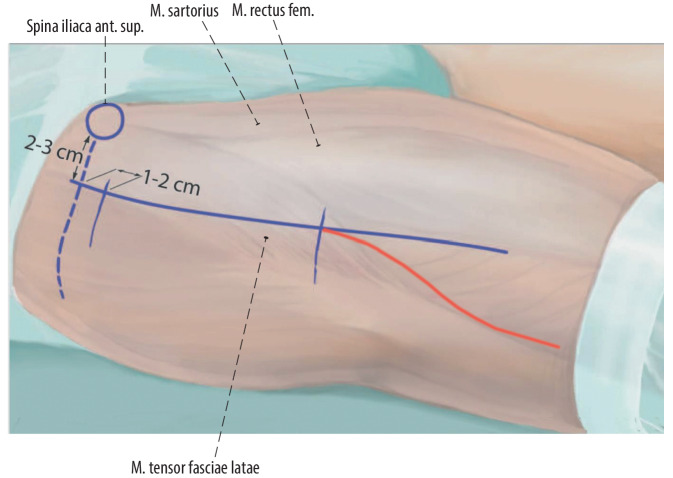
The anterior superior iliac spine (ASIS) is marked; about 2–3 cm lateral to this point and about 1–2 cm distal to it, the skin incision can be started. The length of the skin incision is about 7–8 cm. The skin incision can of course be longer. However, we observed that a length of more than 11 cm has no advantages in standard cases. The skin incision can be extended any time proximally or distally if needed. A proximal extension of the skin incision beyond the ASIS line is not recommended as the muscles are attached in this area and they represent an inner natural barrier. The *red line* shows the Lazy S skin incision extension for the approach to the femoral diaphysis. When releasing the tensor fasciae latae (TLF) muscle from the iliac crest, it is necessary to extend the incision to the ASIS. We strongly recommend to perform a release of the TFL muscle for the treatment of femoral neck fractures with very osteoporotic bone. This allows for straight access to the femoral canal and reduces the lever arm moment against the greater trochanter, thus, reducing the risk of greater trochanter fractures. The wound is carefully expanded with two wound retractors, and the subcutaneous fat tissue is visible. This is especially important for patients with a thicker fat tissue in order to clearly identify the anatomical structures in this small wound window. In all small approaches, the presentation of the anatomical structures is very important to clearly see the main landmarks. The subcutaneous fat tissue is gently dissected until the muscle fasciae are visible. (Adapted from [14])

**Fig. 5:**
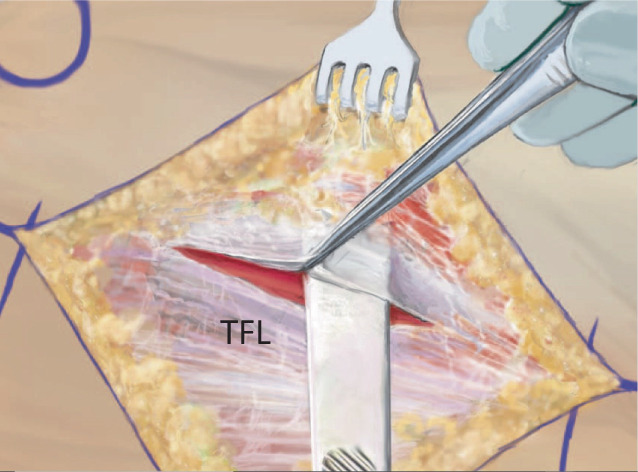
After the careful dissection of the subcutaneous fat layer, the tensor fasciae latae (*TFL*) muscle should be visible anterior to the white fascia, the part of the iliotibial tract which covers the gluteus medius muscle. At the lower margin of the TFL muscle, perforating vessels [18] can usually be found in this region. As soon as the TFL muscle is identified, the fascia of the TFL muscle is incised at its midpoint, the fascia is lifted with forceps and a blunt retractor is placed underneath the fascia. Preparation underneath the fascia is important to avoid direct or indirect injury to the lateral femoral cutaneous nerve. It is not recommended to search these nerves as scars can occur in this area which might lead to a nerve compression and to the very painful meralgia paresthetica. (From [14])

**Fig. 6:**
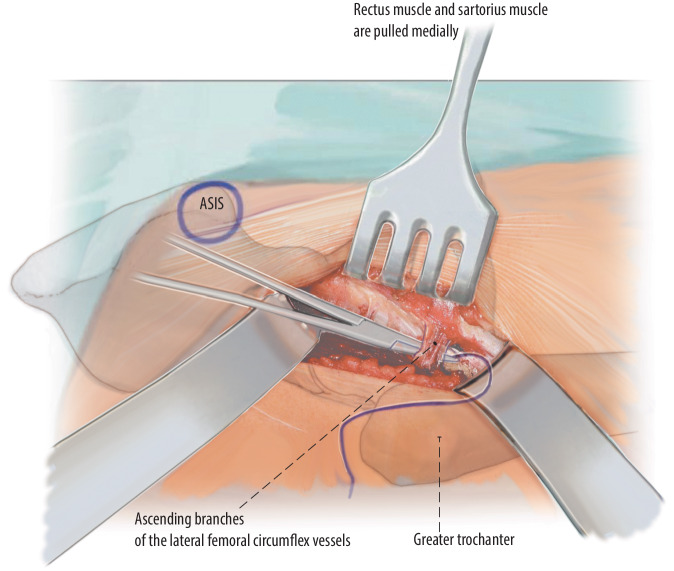
The muscle fascia medial to the fibers of the tensor fasciae latae muscle is split with the finger. Proximal to it, in the area of the upper wound margin, a curved retractor is introduced, its tip is oriented toward the ilium and the capsule. The fascia is then opened distally with the finger in order to place the second retractor. This second retractor is placed on the distal wound margin leaving its tip at the proximal femur at the level of the greater trochanter. With a deep broad wound retractor, the rectus muscle and the sartorius muscle are pulled medially, revealing the vessels. These vessels are the muscular ascending branches of the lateral femoral circumflex vessels. They need to be carefully ligated. From distal, strong tendon fibers can lead into the capsule; this is the deep layer of the iliotibial tract. To reach the capsule, they need to be cut [16]. The precapsular fat pad should be pushed aside in order to dissect with a Cobb elevator medially around the neck of the prosthesis. In primary approaches, this fat pad is always present. *ASIS* anterior superior iliac spine

**Fig. 7:**
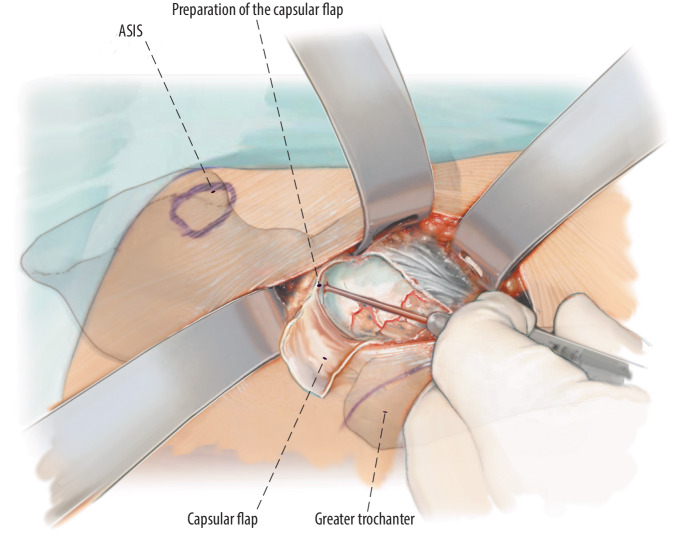
Once the medial side of the femoral neck is reached, a curved retractor is inserted which pulls the rectus femoris muscle medially. Another retractor is placed in the same manner at the cranial rim of the acetabulum. The curved retractor is pushed around the rim of the acetabulum and is held in place. The retractor needs to be inserted dorsal to the iliopsoas muscle in order to avoid injury to the femoral nerve and the femoral vascular bundle. The orientation of the tip needs to be perpendicular to the inguinal ligament. *ASIS* anterior superior iliac spine

**Fig. 8:**
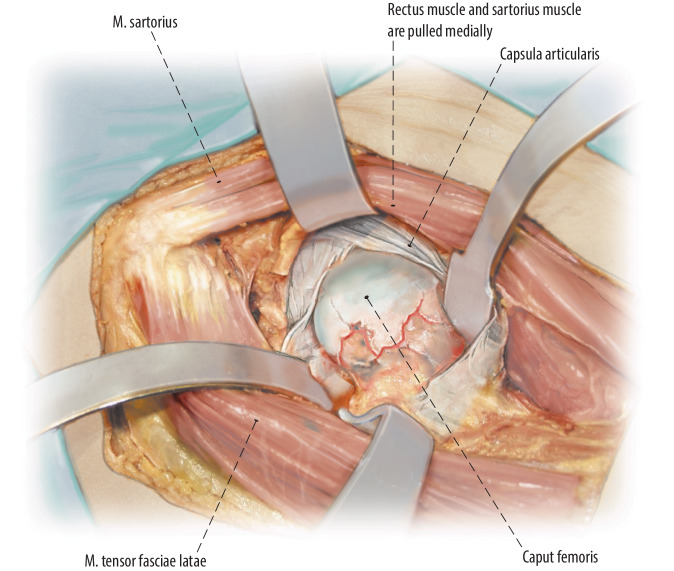
An H‑shaped incision of the capsule is made. We make sure that the capsule under the iliopsoas muscle remains intact. The capsule layer represents a protection against the instruments during the preparation of the bone. Laterally we form a capsule flap which can be refixed if desired

**Fig. 9:**
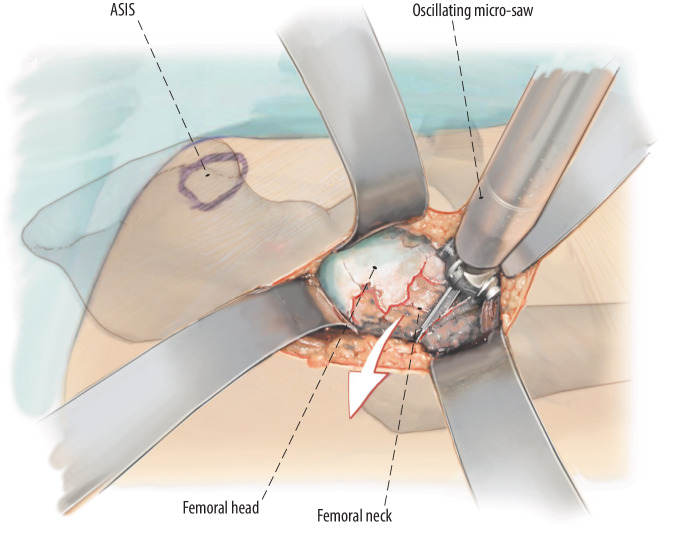
After opening the capsule, the typical hematoma is irrigated after bacteriological samples have been taken. The femoral neck is surrounded by two blunt retractors. The fracture is now inspected. Remaining parts of the femoral neck are removed. The final osteotomy at the femur is performed with a saw, and a second osteotomy is performed at the lower rim of the femoral head. The remaining bone from the femoral neck is completely removed. A corkscrew is inserted in the femoral head and the femoral head is removed. Make sure to not damage the muscle fibers with the sharp cutting edge of the femoral head or other fragments when removing the head. *ASIS* anterior superior iliac spine

**Fig. 10:**
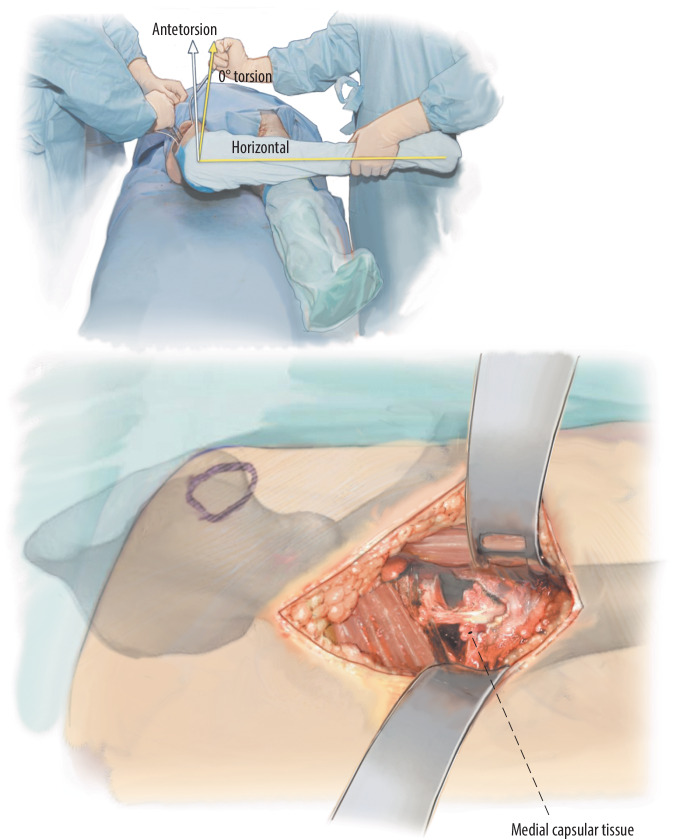
Then the leg is placed in a figure-of-four position. In this way, the femur is externally rotated and the medial part is positioned upwards. The capsule which still attaches at the medial femoral neck is detached if it impedes the external rotation. The lesser trochanter is palpated and the height of the osteotomy is verified and re-osteotomized where necessary. In the figure-of-four position, the calcar shows upwards, the retrocondylar plain is in the vertical position. Hence, the vertical axis indicates 0° of torsion of the shaft. This 0° can be marked on the bone as a reference point when adjusting the torsion of the shaft [11]

**Fig. 11:**
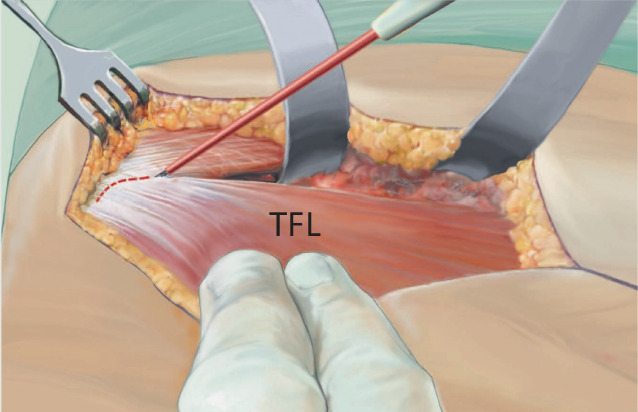
In many instances, with elaborate instrumentation and shorter implants it is possible to avoid any muscular releases. Yet, if the situation is more challenging, an additional release is still possible in our experience. Therefore, to avoid a damage of the greater trochanter in patients with osteoporotic bone, it is recommended to perform a release of the tensor fasciae latae (TFL) muscle. To this end, the skin incision is proximally extended to the anterior superior iliac spine (ASIS). About 1 cm distal to the ASIS, the TFL muscle is incised. Make sure that the tractus is also incised at this point. This release allows the femoral retractor to be pushed more dorsally and to have straight access to the femoral shaft. (From [14])

**Fig. 12:**
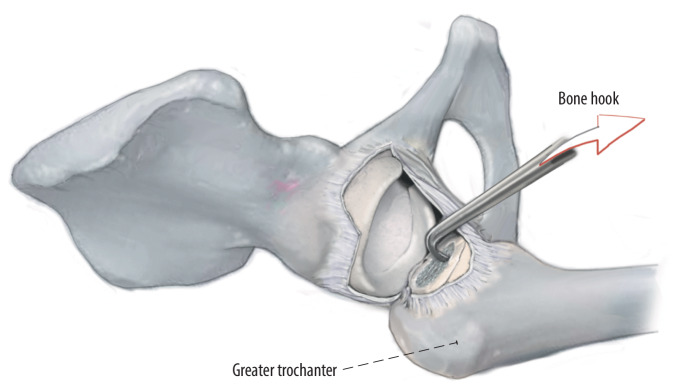
After the incision of the fasciae latae, a hook is placed into the femur in order to pull it ventrally. The femoral elevator, a double-pronged retractor, is placed at about 25° to the femoral axis dorsal to the greater trochanter with the leg externally rotated. By pulling the hook and pushing the femoral elevator at the same time, the femur can be elevated in order to have straight access to the femoral canal. A medial retractor can be placed in order to hold back the soft tissue medially

**Fig. 13:**
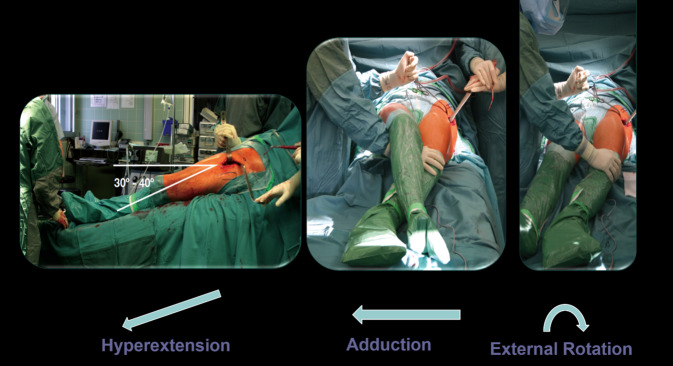
In order to achieve good exposure of the proximal femur, the position of the leg is very important. External rotation, hyperextension and adduction need to be achieved. Hyperextension is achieved by breaking the table and lowering the leg part by 30–40°. The table joint should be at the level of the greater trochanter. The operated leg is adducted either by crossing it under the opposite leg or by additionally abducting the opposite leg. No post or other fulcrum is used. According to our experience, it is not necessary to pull the leg. It is also not necessary to achieve extreme positions. With an extension table it is absolutely not recommended to use excessive force. Depending on the muscular situation of the patient, it is necessary to find for each case the best position. *Caution*: The knee should be fully extended. Every flexion of the knee leads to a higher tension of the proximal part of the quadriceps and therefore to a diminution and a narrowing of the approach

**Fig. 14:**
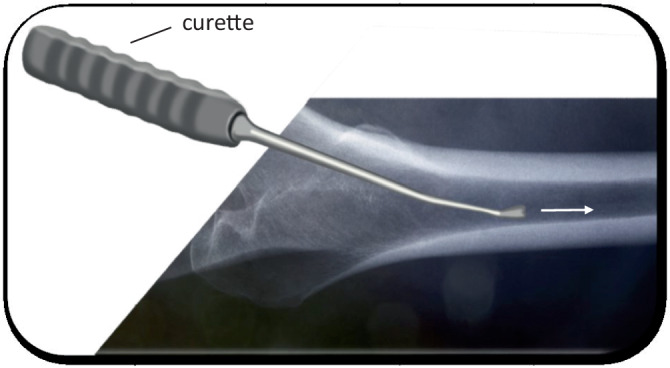
For safe preparation of the femur, it is essential to open the femoral canal with simple curved instruments. We start with the simple curved curette which is inserted into the femoral canal. The curette is sharp enough to slide through the soft spongiosa and blunt enough at the same time at the outside in order to avoid injury and perforation of the corticalis. Then the proximal opening of the canal is extended with a Luer until the shape and size of the planned femur prosthesis is reached. Usually there is still a lot of corticalis left at the femoral neck. It should be removed now in order to be able to bring the shaft more lateral (lateral in relation to the femur according to the present position, it means more posterior!). Some systems (Accolade II, Exeter, Stryker, Mahwah, NJ, USA) have the small opening broaches which we recommend. They can now be hammered in, in order to reach the correct form of the proximal prosthesis shaft. As alternative, an opening box chisel can be used. Independent from the system used, it is mandatory to use a broach handle with double-offset (anterior and lateral), which allows in all cases a gentle preparation and introduction of the rasp around the soft tissue [13, 17]

**Fig. 15:**
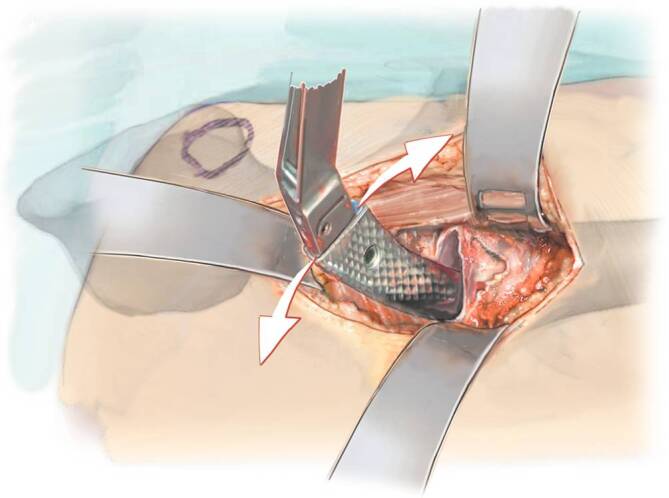
Now the smallest available broach is attached to the handle. Insert the broach carefully and with constant vibrating up and down movements of the broach handle without using a hammer. Once the rasp is fully inserted, the orientation inside the shaft is achieved, and a perforation of the bone is not possible anymore, a mallet can be used. When the final orientation is achieved, the rasp can also be hammered in until the right depth is achieved. All other rasps are inserted in the same manner, until the final size has been achieved. The figure shows that the broach handle is parallel to the axis of the femur once the prosthesis is inserted deep enough into the canal. The hammer should only be used in this position in order to avoid perforation of the femur

**Fig. 16:**
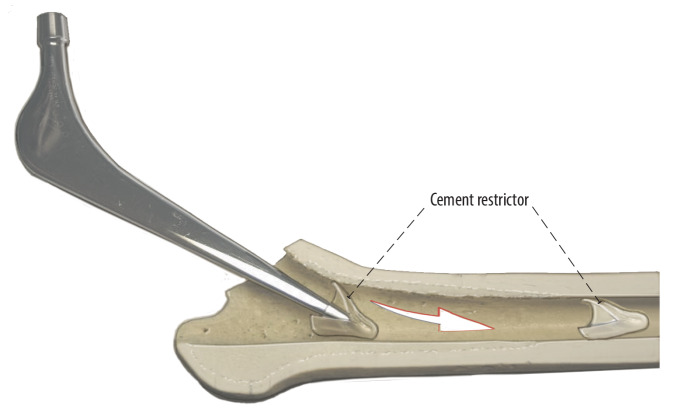
The prosthesis is inserted in a curved way. We could show in a study that this has no negative influence on the cement mantle [7, 9]. A cement restrictor is used as well as a fourth generation cementing technique. Once the cemented or uncemented shaft is inserted, it can be brought to the right depth with a simple straight inserter with a blunt end

**Fig. 17:**
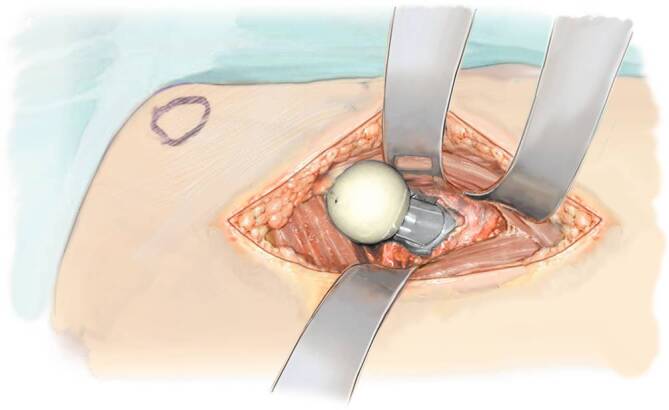
Trial positioning is done with a dual head system after final polymerization of the cement. The size of the head is based on preoperative planning with a digital x‑ray templating system (MediCAD. HECTEC, Altdorf, Germany) and double checked with head trials. The final head is inserted and fixed with soft hammer strokes. The hip is now reduced. In some cases, it might be easier to first introduce the dual head into the acetabulum and then to reduce the neck of the prosthesis into the head. Then, if desired, the capsule is closed. We recommend infiltration of the area of the lateral femoral cutaneous nerve and the subcutis with a local anesthetic to reduce pain

**Fig. 18:**
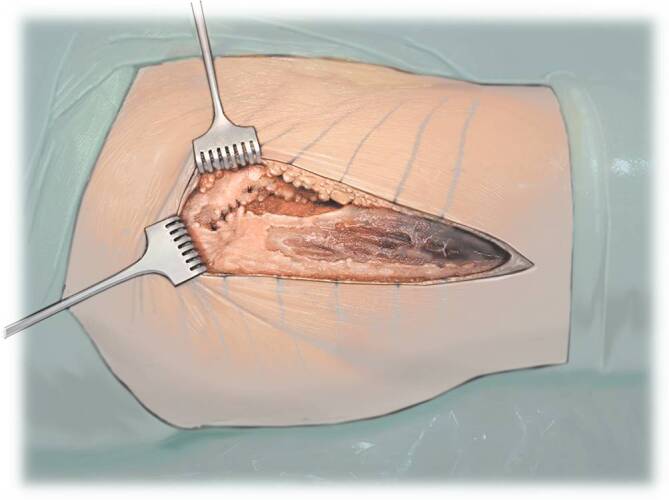
Reattachment of tensor to the bone. The fascia over the tensor fasciae latae muscle is sutured with a continuous matrix suture. As the capsule has been partially removed, the remaining part is left as it is. We do not insert any drains. The wound is also sutured with a continuous intracutaneous resorbable suture. Therefore, the suture does not need to be removed. The wound is closed with a silver impregnated wound dressing

## Postoperative management

Early mobilization is mandatory for older patients. This should be started as early as possible after the surgery. The technique allows the patient to immediately mobilize. Full weight bearing can be allowed depending on the stem fixation and the weight of the patient. In cemented prosthesis, weight bearing is unrestricted. We have observed very little pain in our patients postoperatively. Therefore, we believe the DAA supports the need for early mobilization optimally.

## Errors, hazards, complications

- *Reduction problem:* Sometimes it is difficult to reduce the large head underneath the rectus tendon. In such cases, we recommend placing a curved retractor under the tendon around the anterior acetabular rim. It is sometimes also helpful to place the head into the acetabulum first and to reduce the neck into the head afterwards.
- *Femur fractures:* As in all hip arthroplasties, specifically in weak bone stock, fracture of the femur can occur [12] and adequate cabling or wiring equipment should be available. A distal extension of the approach is possible in cases of femoral fractures [20]
- *Greater trochanter fractures: *Fractures of the greater trochanter can be avoid by careful handling and the TFL release in most cases. If one occurs we do not treat fracture of the tip of the greater trochanter and fractures with very bad bone stock of the fragment. Lager fragments can be refixated with clamps. In order to place the clamp we perform a second revision strictly from lateral latera in order to insert the clamp. Cabling is performed through the anterior portal.
- *Obesity:* In very obese patients, special care should be taken in wound treatment postoperatively. Repeated wound disinfection should be performed postoperatively.
- *Potential lesion of the lateral femoral cutaneous nerve:* with numbness at the distal lateral femur.

## Results

The direct anterior approach (DAA) was retrospectively compared versus other approaches in hemiarthroplasty for femoral neck fractures in patients over 80 years of age at the KAT hospital in Athens.

From January 2010 until July 2019, 1158 femoral neck fractures were treated with hemiarthroplasty in the hospital. The posterior approach was used in 656 cases, Hardinge approach in 312 cases, 116 cases were treated with DAA and 74 with anterolateral approach. Data regarding dislocations, periprosthetic fractures, blood loss, postoperative pain, duration of surgery, postoperative mobility and length of stay were recorded and retrospectively analyzed.

Table 1 presents the demographics and the characteristics of the patients according to each approach. Gender and age did not differ between groups and follow-up was longer for posterior and Hardinge approaches, as they were the most commonly performed approaches at the beginning of the study. The age range was 80–103 years and no significant differences regarding age were observed between groups.

**Table 1:** Demographics and patients’ characteristics

	Posterior (*n* = 656)	Hardinge (*n* = 312)	DAA (*n* = 116)	Anterolateral (*n* = 74)
*Gender, Male/Female; n (%)*	312 (47.6%)/344 (52.4%)	164 (52.6%)/148 (47.4%)	52 (44.8%)/64 (55.1%)	36 (48.6%)/38 (51.4%)
*Age (years)*	89.13 ± 8.24	86.64 ± 7.44	85.32 ± 4.32	84.73 ± 3.96
*Follow-up (years)*	5.24 ± 1.77	5.69 ± 2.79	3.16 ± 1.25	3.64 ± 1.32

*DAA* direct anterior approach

All of the patients had similar comorbidities according to their advanced age and no significant differences were observed among the groups.

Patients were optimized preoperatively. If hemoglobin was less than 9 mg/dl patients were transfused, according to the protocol of our clinic. Blood loss was calculated based on the need for transfusion in the early postoperative period and the hemoglobin drop between preoperative and 1st postoperative day values.

We used the VAS (visual analogue scale) score to quantify pain which was measured at the 2nd postoperative day. For the DAA a traction table was used with patient in supine position. The other approaches were performed in the lateral decubitus position.

Table 2 presents intra- and postoperative periprosthetic fractures and also dislocations between the four groups. The dislocation rate seems similar among groups; however, when subgroup categories were analyzed, patients operated with DAA had significantly lower dislocations in comparison to those operated with posterior approach (13.3% vs 1%), as shown in Table 3.

**Table 2:** Complications between groups

	Posterior (*n* = 656)	Hardinge (*n* = 312)	DAA (*n* = 116)	Anterolateral (*n* = 74)
*Dislocation*	8 (1.2%)	3 (0.96%)	1 (0.86%)	1 (1.35%)
*PPF intra-op*	6 (0.9%)	4 (1.28%)	2 (M.T. avulsion) (1.7%)	2 (M.T. avulsion) (2.7%)
*PPF post-op*	15 (2.28%)	8 (2.56%)	1 (0.86%)	1 (1.35%)

*PPF* periprosthetic fractures, *DAA* direct anterior approach, *M.T.* musculus tenosr fasciae latae avulision

**Table 3:** Subgroup categories between posterior and DAA in patients with neurological diseases

	Posterior (*n* = 45/656)	DAA (*n* = 98/116)	*p*-value
*Dislocation*	6 (13.3%)	1 (1%)	< 0.001

*n* patients with neurological disease (mainly Parkinson’s and dementia)/total cases, *DAA* direct anterior approach

We mobilize patients with weight bearing as tolerated at the 1st postoperative day. Those patients treated with DAA performed better regarding walking as they felt more confident due to better muscular tone.

In all patients, postoperative pain was managed with dexketoprofen and acetaminophen. Patients treated with DAA reported less postoperative pain compared to other groups (VAS score). In patients with dementia the evaluation of pain was difficult.

Blood loss was less in the DAA and anterolateral approach (2.22 and 2.23 g/dL) than in posterior and Hardinge approaches (3.25 and 3.35 g/dL), according to hemoglobin drop count.

Patients treated with DAA and anterolateral approach were discharged on the 2nd postoperative day and on the 4th day for the other approaches, with the exception of the patients who developed complications, either surgical or medical.

Data regarding blood loss, postoperative pain, duration of surgery, and length of stay are summarized in Table 4.

**Table 4:** Data regarding blood loss, pain, duration of surgery and length of stay

	Posterior (*n* = 656)	Hardinge (*n* = 312)	DAA (*n* = 116)	Anterolateral (*n* = 74)
*Pre-op transfusion*^*a*^	15	8	3	2
*Post-op transfusion*^*a*^	9	5	1	1
*Hb drop*^*b*^	3.25 ± 1.16	3.35 ± 1.01	2.23 ± 1.21	2.22 ± 1.03
*Post-op pain (VAS score)*	57.3 ± 23.6	58.1 ± 21.4	28.3 ± 16.4	33.2 ± 20.5
*Duration of surgery (min)*	31–47	30–48	30–45	30–46
*Length of stay (days)*	4.2 ± 2.3	4.8 ± 2.4	2.3 ± 1.6	2.8 ± 1.4

*DAA* direct anterior approach, *VAS* visual analoge scale, *Hb* hemoglobine

^a^Number of patients needed RBC transfusion

^b^This is the calculated difference between pre-operative and 1st postoperative day expressed in g/dL

Our results indicate that patients treated with anterior approaches had less postoperative pain, less blood loss, and reduced hospital stay than those treated with posterior or Hardinge approach. Surgical duration and comorbidities had no significant differences among the groups. Appropriate set-up with the positioning of the patients on traction table and draping of patients in DAA did not add extra time to the duration of surgery as it is a regularly performed approach in our center.

We regularly use DAA or anterolateral approach to treat patients with neurological diseases, as they were associated with less postoperative pain, less blood loss, faster and safer mobilization. It is very important in these patients to pay attention to the positioning of the stem in order to avoid excessive anteversion, as they tend to keep their hip in flexion, abduction and external rotation increasing the risk of dislocation.

## Declarations

All studies performed were in accordance with the ethical standards indicated in each case.
